# Normal childbirth: The natural, non-medical, alternative approaches to the most common medical interventions in labor

**DOI:** 10.18332/ejm/174525

**Published:** 2023-12-01

**Authors:** Dimitrios Papoutsis, Angeliki Antonakou

**Affiliations:** Department of Midwifery, School of Health Sciences, University of Western Macedonia, Ptolemaida, Greece; Department of Midwifery, School of Health Sciences, International Hellenic University, Thessaloniki, Greece

**Keywords:** normal childbirth, medical interventions, natural methods, intrapartum care

The World Health Organization (WHO) has published over the years several reports addressing the issues of care in normal birth, irrespective of the setting, the level of care or country^1,2^. It has examined the evidence on many of the commonest intrapartum practices and has made recommendations about those interventions that should be utilized to support the processes of normal birth. A seminal report was the WHO definition in 1996 of ‘normal birth’ as the birth that is of spontaneous onset, with gestational age of 37 to 42 weeks of pregnancy, and the baby being born in a vertex position. What is most important is that the WHO definition continued stating that the aim of a normal birth is a healthy mother and child, with the least number of interventions, and that there should be valid reasons for interfering with the natural process of birthing. Since this report in 1996, childbirth in both developed and developing countries has moved away from the concept of normality and the least possible level of interventions and has become significantly medicalized^3^.

If we were to support normality at childbirth and to reduce the number of medical interventions, we probably need to revisit our intrapartum care practices and philosophy of care^2,4^. We need to further question the existing evidence-base behind our intrapartum interventions, some of which have become routine, such as the regular vaginal examinations we perform in labor, that they are not even considered as an intervention^5^.

In most high-income countries most women give birth in maternity hospitals, even if they have a low-risk pregnancy^6^. Among the most common intrapartum interventions that pregnant women are very likely to receive is regular vaginal examinations, the intravenous use of oxytocin for labor augmentation, the use of an epidural analgesia for labor pain relief, restricted or no mobility during labor, and giving birth in a lithotomy position. Nevertheless, there is now abundant evidence in the literature demonstrating that for every intrapartum medical intervention there is a clinically equivalent natural approach leading to the same desired effect, provided that there is no urgent indication to expedite delivery.

It has been reported that women receive, on average, three vaginal examinations during labor, with the maximum number of examinations as high as seven in some cases^7^. Because vaginal examinations may be painful and may cause emotional distress and embarrassment, and in order to promote normality at birth while at the same time evaluating the progress of labor in an objective way, the WHO and the National Institute for Health and Care Excellence (NICE) recommend that a vaginal examination should be performed every four hours during the first stage of labour^8^.

Oxytocin is the most frequently used medication intrapartum to increase uterine activity and is indicated in cases of prolonged labor or dystocia^8,9^. Nevertheless, there are high rates of misuse of oxytocin identified in the literature for the purpose of shortening the duration of labor, with the result of many babies suffering asphyxia at birth^10^. The nonmedical and natural alternatives to intravenous oxytocin can easily be elucidated when considering the neurobiological basis and the hormonal blueprint of an undisturbed labor and birth^11,12^. It is known that psychological stress and pain can have a negative effect on the progress of labor, as they inhibit the release and effective action of endogenous oxytocin^11^. Moreover, when a pregnant woman mobilizes and assumes upright positions during labor, gravity assists the fetal head descent deeper in the pelvis and supports the proper and even application of the fetal head on the cervix, which in turn activates the Ferguson reflex and leads to additional release of endogenous oxytocin^11,13^. If we were to replace intravenous oxytocin with a natural alternative, we would have to mobilize women during labor and have them assume upright positions and use natural methods, such as the use of water immersion in labor, to relieve stress and pain. There is evidence that women who were mobilized and assumed upright positions, as opposed to lying down on the bed, had a first stage of labor that was shorter by about one hour and 22 minutes^13^. Other reports have found that water immersion may shorten the duration of the first stage of labor by 42 minutes (95% CI: 3.4–89.9), on average^14^.

It has been reported that epidural analgesia is the most effective form of pain relief in labor, with approximately 30% of laboring women in the UK and 60% in the USA receiving epidural analgesia^15,16^. It is considered the gold standard against which all the other non-pharmacological methods of pain relief are compared. Despite its high effectiveness in managing labor pain, it is an intrusive method nevertheless and it has been associated with a prolonged second stage of labor, with a higher occurrence of malpresentations and higher percentage of assisted vaginal births^16^.

Natural approaches that may act as an alternative to an epidural analgesia with a high magnitude of effect size involve water birthing and the use of hypnosis in labor. Water birthing has been found to significantly reduce, by 83%, the need for epidural analgesia (OR=0.17; 95%CI: 0.05–0.56) and thus is often referred to as ‘aquadural’ or ‘wet epidural’^16-18^. Moreover, the use of hypnosis during labor was found to reduce, by 70%, the need for epidural analgesia (RR=0.30; 95% CI: 0.22–0.40)^19^. The need for an epidural was reduced by about 19% (RR=0.81; 95%CI: 0.66–0.99) when women were ambulating and in an upright position during labour^1^.

Ambulation and maintaining an upright position such as kneeling, squatting or standing during the first stage of labor, are critical as they are a natural method of pain relief and also act as a stimulant of endogenous oxytocin release^13^. Studies have shown that if women were allowed to assume any position they wished during labor, then they would opt to mobilize and change positions with an average of 7 to 8 positions^20^.

Furthermore, giving birth in an alternative birthing position, and not in a lithotomy position, has been shown to ease delivery and to facilitate the birthing process as it allows greater coccyx movement and widens the diameter of the bony pelvis^21,22^.

There are several other clinical practices still in use today during labor and birth that have been refuted by the WHO in its 2018 recommendations for intrapartum care and it has been strongly suggested that they should be withdrawn^2^. Practices that should be eliminated involve the routine pubic shaving and use of enema, the routine manual exploration of the uterus after delivery, and the application of manual fundal pressure (known as the Kristeller maneuver) to facilitate childbirth during the second stage of labor. In addition, it is not recommended to restrict oral fluids and food intake in low-risk women, as there is no evidence to support this^2^.

As previously described, there are several non-medical approaches to some of the most common medical interventions that pregnant women endure during labor and birth. These alternative natural approaches that we have described need to be embraced and integrated in a respective philosophy of care. The birth accoucheurs need to understand that labor and birth are profound experiences that carry a significant meaning for women, and therefore intrapartum clinical practices need to be woman-centered, respectful, evidence-based, and genuine^23,24^. Childbirth should be one of the most transformative and rewarding events in a woman’s life. For this reason, she should be provided with both medical and non-medical (natural) options during the birthing process to ensure her safety and that of her baby, clinical effectiveness, and high quality of care.

## Data Availability

Data sharing is not applicable to this article as no new data were created.

## References

[1] Care in normal birth: a practical guide (1997). Technical Working Group, World Health Organization. Birth.

[2] World Health Organization (2018). WHO recommendations Intrapartum care for a positive childbirth experience.

[3] Johanson R, Newburn M, Macfarlane A (2002). Has the medicalisation of childbirth gone too far?. BMJ.

[4] Oladapo OT, Tunçalp Ö, Bonet M (2018). WHO model of intrapartum care for a positive childbirth experience: transforming care of women and babies for improved health and wellbeing. BJOG.

[5] Shepherd A, Cheyne H, Kennedy S, McIntosh C, Styles M, Niven C (2010). The purple line as a measure of labour progress: a longitudinal study. BMC Pregnancy Childbirth.

[6] Scarf VL, Rossiter C, Vedam S (2018). Maternal and perinatal outcomes by planned place of birth among women with low-risk pregnancies in high-income countries: A systematic review and meta-analysis. Midwifery.

[7] Shepherd A, Cheyne H (2013). The frequency and reasons for vaginal examinations in labour. Women Birth.

[8] Intrapartum Care: Care of healthy women and their babies during childbirth (2014). Clinical Guideline 190.

[9] Hidalgo-Lopezosa P, Hidalgo-Maestre M, Rodríguez-Borrego MA (2016). Labor stimulation with oxytocIn: effects on obstetrical and neonatal outcomes. Rev Lat Am Enfermagem.

[10] Oláh KSJ, Steer PJ (2015). The use and abuse of oxytocin. The Obstetrician & Gynaecologist.

[11] Walter MH, Abele H, Plappert CF (2021). The Role of Oxytocin and the Effect of Stress During Childbirth: Neurobiological Basics and Implications for Mother and Child. Front Endocrinol (Lausanne).

[12] Nash K (2020). Physiology’s role in labour assessment. Br J Midwifery.

[13] Lawrence A, Lewis L, Hofmeyr GJ, Styles C (2013). Maternal positions and mobility during first stage labour. Cochrane Database Syst Rev.

[14] Cluett ER, Burns E, Cuthbert A (2018). Immersion in water during labour and birth. Cochrane Database Syst Rev.

[15] Osterman MJK, Martin JA (2011). Epidural and Spinal Anesthesia Use During Labor: 27-state Reporting Area, 2008. Natl Vital Stat Rep.

[16] Halliday L, Nelson SM, Kearns RJ (2022). Epidural analgesia in labor: A narrative review. Int J Gynaecol Obstet.

[17] Burns E, Feeley C, Hall PJ, Vanderlaan J (2022). Systematic review and meta-analysis to examine intrapartum interventions, and maternal and neonatal outcomes following immersion in water during labour and waterbirth. BMJ Open.

[18] Halek JE (2000). Aquadurals and douladurals replace the epidurals. Midwifery Today Int Midwife.

[19] Smith CA, Collins CT, Cyna AM, Crowther CA (2006). Complementary and alternative therapies for pain management in labour. Cochrane Database Syst Rev.

[20] Smith MA, Acheson LS, Byrd JE (1991). A critical review of labor and birth care. Obstetrical Interest Group of the North American Primary Care Research Group. J Fam Pract.

[21] Borges M, Moura R, Oliveira D, Parente M, Mascarenhas T, Natal R (2021). Effect of the birthing position on its evolution from a biomechanical point of view. Comput Methods Programs Biomed.

[22] Satone PD, Tayade SA (2023). Alternative Birthing Positions Compared to the Conventional Position in the Second Stage of Labor: A Review. Cureus.

[23] (2019). Strengthening quality midwifery education for Universal Health Coverage 2030: Framework for action.

[24] Bradfield Z, Hauck Y, Kelly M, Duggan R (2019). “It’s what midwifery is all about”: Western Australian midwives’ experiences of being ‘with woman’ during labour and birth in the known midwife model. BMC Pregnancy Childbirth.

